# Supplementary material for: The Cluster Transfer Function of AtNEET Supports the Ferredoxin–Thioredoxin Network of Plant Cells
Source: Antioxidants (Basel). 2022 Aug 6;11(8):1533. doi: 10.3390/antiox11081533 (PMC9405330; doi:10.3390/antiox11081533)
Supplement: Supplementary file 1 [file antioxidants-11-01533-s001.zip › antioxidants-1808438-supplementary.pdf]

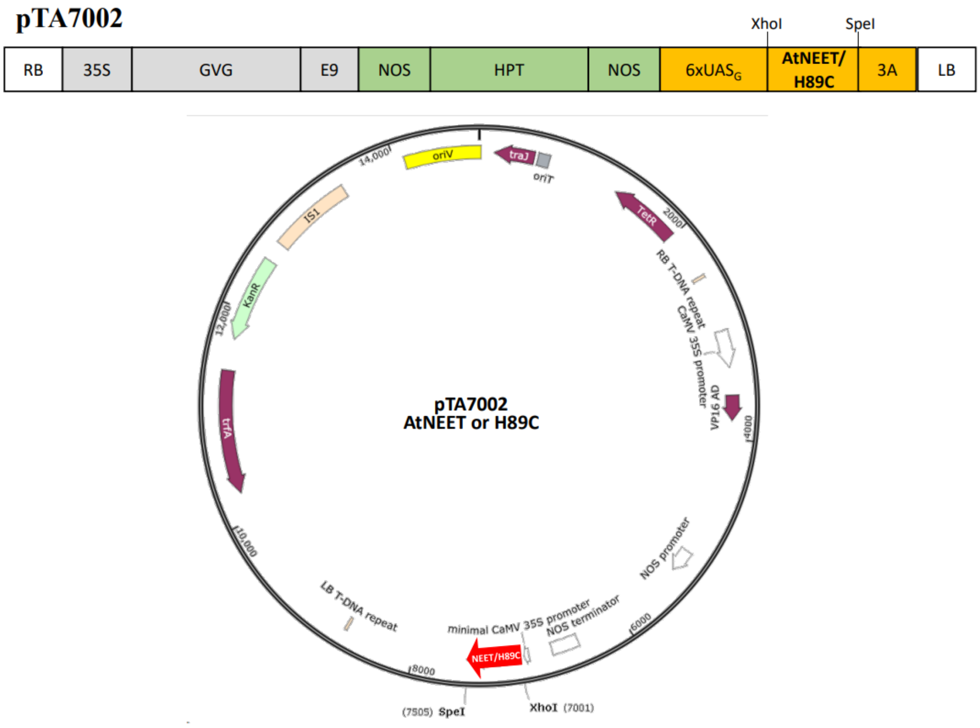

Figure S1. The dexamethasone (DEX)-inducible system to drive the expression of AtNEET, or its mutated dominant-negative copy H89C, in mature transgenic Arabidopsis plants. AtNEET or H89C were amplified and cloned into pTA7002 vector [12] using XhoI and SpeI sites. Abbreviations used: RB, right border; 35S, cauliflower mosaic virus 35S promoter; E9, the poly(A) addition sequence of the pea ribulose bisphosphate carboxylase small subunit rbcs-E9; GVG, chimeric transcription factor GVG composed by the heterologous DNA-binding of the yeast transcription factor GAL4, the transactivating domains from the herpes viral protein VP16, and the glucocorticoid receptor (GR); NOS, nopaline synthase promoter and terminator; HPT, hygromycin phosphotransferase; 6xUASG, six copies of the GAL4 UAS; 3A, the poly(A) addition sequence of the pea rbcS-3A; LB, left border.

TRXs

		Line 1	Line 2	
	AtNEET	0,90	0,75	AT1G03680 TRX M-type 1
	H89C	n.s.	n.s.	
	AtNEET	0,86	0,75	AT1G21350 TRX superfamily protein
	H89C	n.s.	n.s.	
	AtNEET	n.s.	n.s.	AT1G65970 TPX2
	H89C	4,86	1,28	
	AtNEET	0,77	0,76	AT2G04700 Fd-TRX reductase catalytic beta chain
	H89C	n.s.	n.s.	
	AtNEET	0,75	0,67	AT3G02730 TRX F-type 1
	H89C	0,75	0,81	
	AtNEET	n.s.	n.s.	AT3G11630 TRX superfamily protein
	H89C	1,12	1,10	
	AtNEET	n.s.	n.s.	AT1G65980 TPX1
	H89C	2,51	1,16	
	AtNEET	n.s.	n.s.	AT1G76020 TRX superfamily protein
	H89C	0,51	0,76	

APXs

	Line 1	Line 2	
AtNEET	0,82	0,86	AT4G08390 Stromal APX
H89C	1,40	n.s.	
AtNEET	n.s.	n.s.	AT4G09010 APX4
H89C	0,80	0,70	

GPXs

	Line 1	Line 2	
AtNEET	0,62	0,80	AT4G11600 GPX6
H89C	1,78	n.s.	

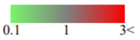

Figure S2. Changes in steady state expression of different transcripts involved in reactive oxygen species (ROS) scavenging reported previously [9] in two different lines with constitutive expression of AtNEET or H89C. Abbreviations used: APX, ascorbate peroxidase; GPX, glutathione peroxidase; n.s., not significant; TRX, thioredoxin.

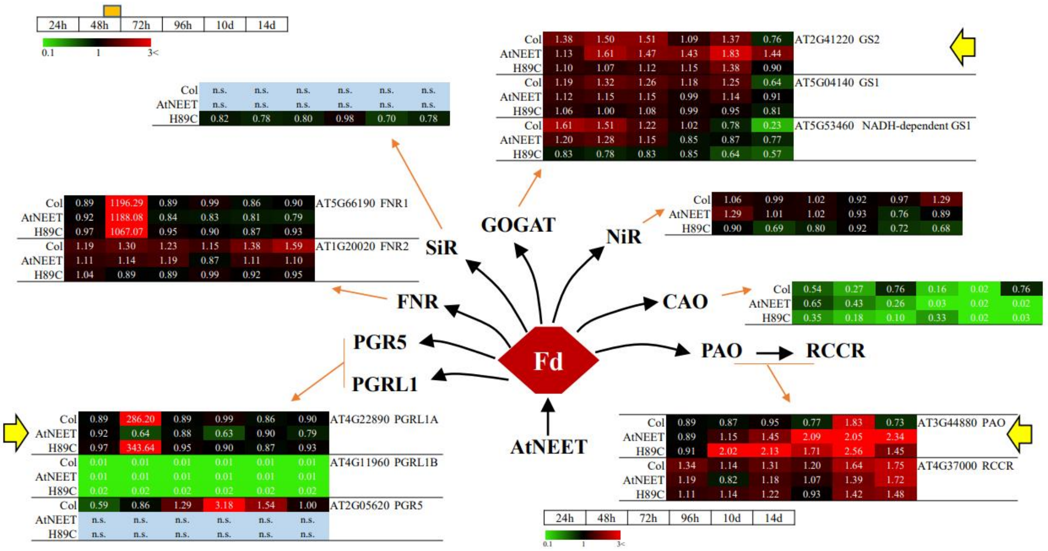

Figure S3. Changes in protein expression associated with other functions of ferredoxins during the course of the experiment. Pathway and heat maps for the expression of different proteins with a significant change in expression (in at least one time point, compared to time 0 h within each genotype) associated with additional ferredoxin functions in Arabidopsis at the different time points are shown. All experiments were repeated at least three times with similar results. Yellow arrows highlight proteins of interest. Abbreviations used: CAO, chlorophyll A oxygenase; Fd, ferredoxin; FNR, ferredoxin-NADP(+)-oxidoreductase; GOGAT, glutamine oxoglutarate aminotransferase; GS, glutamine synthase; NiR, nitrite reductase; PAO, pheophorbide A oxygenase, PGR5, proton gradient regulation 5; PGRL1A, proton gradient regulation 5-like A; PGRL1B, proton gradient regulation 5-like B; RCCR, red chlorophyll catabolite reductase; SiR, sulfite reductase.

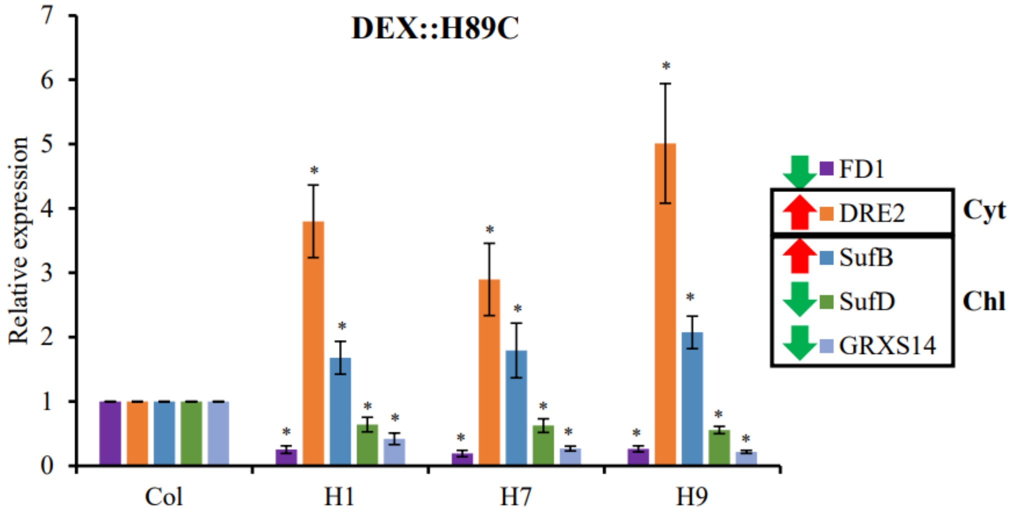

Figure S4. Changes in steady state expression of different transcript associated with iron-sulfur cluster assembly in the chloroplast and cytosol in three different homozygous H89C lines (H1, H7 and H9) following 4 doses of DEX application (Fig. 1A). All experiments were repeated at least three times with similar results. Asterisks denote statistical significance with respect to control (Col) at * $P < 0.05$ (Student t-test, SD, N=5). Abbreviations used: Chl, chloroplast; Cyt, cytosol; DEX, dexamethasone; DRE2, Homolog of Yeast DRE2; FD1, ferredoxin1; GRXS14, Glutaredoxin S14; SufB, Sulfur B; SufD, Sulfur D
